# Giant-Cell Ependymoma of the Cervical Spinal Cord With Syringomyelia and Pathological Presentation: A Case Report and Review of the Literature

**DOI:** 10.7759/cureus.33174

**Published:** 2022-12-31

**Authors:** Hoai Thi Phuong Dinh, Hasegawa Tomohiko, Rina T Madelar, Hao Dinh Anh Hoang, Matsuyama Yukihiro

**Affiliations:** 1 Department of Neurosurgery, Hue University of Medicine and Pharmacy, Hue University, Hue City, VNM; 2 Department of Orthopedic Surgery, Hamamatsu University School of Medicine, Hamamatsu, JPN; 3 Department of Orthopedic Surgery, The Medical City, Pasig City, PHL; 4 Department of Orthopedic Surgery, Hue University of Medicine and Pharmacy, Hue University, Hue, VNM

**Keywords:** giant-cell ependymoma, ki-67, spinal cord, ependymoma, syringomyelia

## Abstract

Ependymomas are unusual neuroepithelial tumors of the central nervous system that arise from clusters of ependymal cells. In adults, ependymomas are the most common primary spinal cord tumors. Nevertheless, only a few cases of large-cell ependymoma have been documented; these cases often involve the brain. Here, we report the case of a 43-year-old man who had a cervical spinal cord ependymoma with syringomyelia. The giant-cell ependymoma (GCE) in the spinal cord discussed in this case emphasizes the characteristics of GCE and the discrepancy between the pathological appearance, the surgical results, and the clinically good prognosis.

## Introduction

Ependymomas are infrequent neuroepithelial tumors of the central nervous system (CNS) that develop from clusters of ependymal cells that border the central canal of the spinal cord and the ventricles of the brain. These tumors account for 2-6% of CNS tumors [1] to 60-70% of spinal cord tumors [2]. Ependymomas are divided into the following three grades by the World Health Organization (WHO):, grade 1 includes myxopapillary and subependymoma; grade II includes cellular, papillary, clear cell, and tanycytic; and grade III includes others, namely, anaplastic [3].

Giant-cell ependymomas (GCEs), a rare type first described in 1996, are frequently categorized as those measuring more than 4 cm [4]. Giant-cell glioblastoma, pleomorphic xanthoastrocytoma, subependymal giant-cell astrocytoma, and anaplastic oligodendroglioma are among the tumors that might represent a differential diagnosis for GCE [5].

In this report, we present a case of ependymoma accompanied by syringomyelia, the presentation of the pathogenesis, WHO’s new classification, and the treatment and prognosis for spinal ependymoma.

## Case presentation

A 43-year-old male patient had cervical spine pain two years ago accompanied by numbness of the fingertips. Recently, the numbness of the fingertips appeared more often and had spread to the lower extremities affecting the patient’s activity.

Physical examination revealed the numbness of the left-sided finger and wrist, around the abdomen, and ankles on two sides, biceps reflex +/+, triceps reflex +/+, brachioradialis +/+, Hoffman -/-, patellar tendon reflex ++/++, Achilles tendon reflex ++/++, Babinski-/-, Barre’s sign, Romberg’s sign, and no cerebellar symptoms.

**Figure 1:**
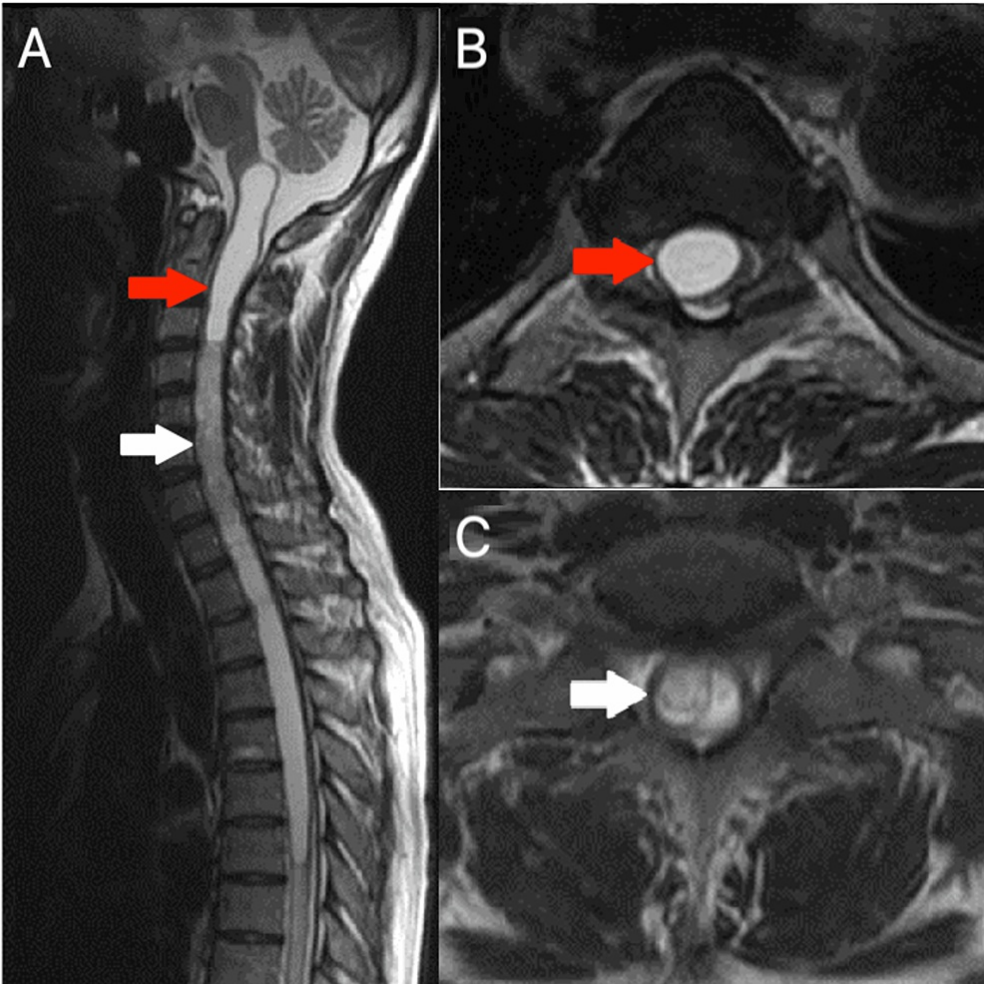
T2-weighted MRI in the sagittal plane (A) shows tumors from C4-C7 (white arrow) with syringomyelia upper and lower tumor (red arrow), corresponding to the axial plane through the syringomyelia (B) and through the tumor with multicystic components (C).

A massive, multicystic, and partly enhancing cervical intramedullary tumor reaching from C4 to C7 was identified using T2-weighted MRI with the sagittal plane. Additionally, there was a syringomyelic cavity that connected the medulla oblongata to T7. On T2-weighted, sagittal images, the syringomyelic cavity and cysts were clearly visible (Figure 1). The C2-T1 laminotomy was performed on the patient.

**Figure 2:**
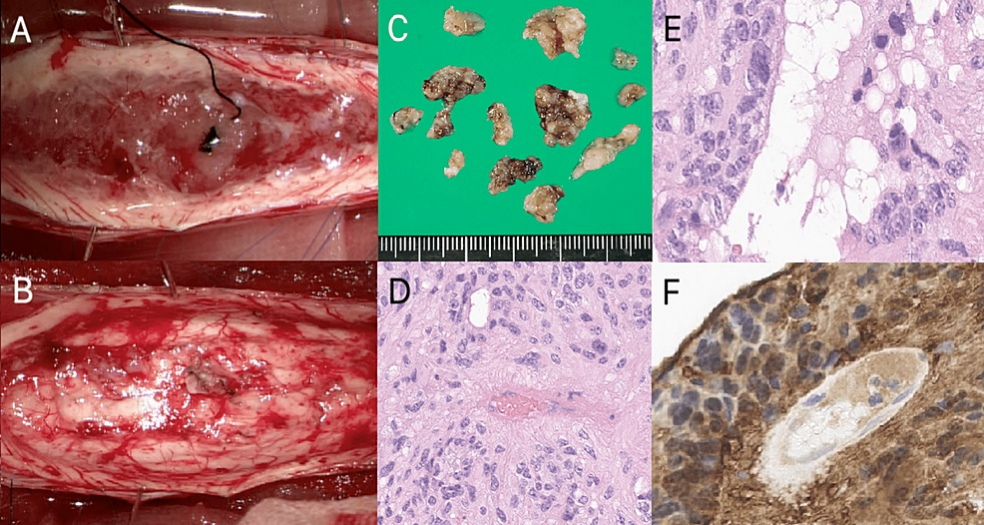
Imaging of the intramedullary tumor during surgery (A), full dissection of the tumor (B), and cutting of the tumor into perivascular pseudorosette for histology (C). (D). The massive cell has eosinophilic cytoplasm, eccentrically positioned single or multiple nuclei with noticeable nucleoli (D), and intranuclear cytoplasmic inclusions (E). The immunohistochemistry stain GFAP revealed the presence of tumor cells (F).

After the myelotomy, a hard mass mixed with gray and yellow colors was noted (Figures 2A, 2B). Macroscopically, its features were consistent with a tumor that was not particularly well infiltrating. Ependymoma was identified in the first few small tissue fragments on frozen-section analysis. The laminar plateau was relocated and fixed with mini plates after the arachnoid was closed, and the dura mater was sutured. Early neurological improvement with decreased finger and toe numbness was seen following surgery. He began rehabilitation therapy, and his neurological condition steadily improved over time. Pathological analysis on hematoxylin and eosin (H&E)-stained paraffin sections (Figures 2C, 2D) showed a moderately cellular tumor with a biphasic pattern. One pattern had perivascular pseudorosettes (Figure 2D). There were also a few ependymal canals with eosinophilic inclusions and tiny columnar cells lining them (Figure 2E). Giant cells and the cells generating perivascular pseudorosettes both substantially expressed GFAP, S-100 protein, and vimentin in the cytoplasm on immunohistochemistry (Figure 2F). The Ki-67 labeling index was about 5.7% in the areas.

**Figure 3:**
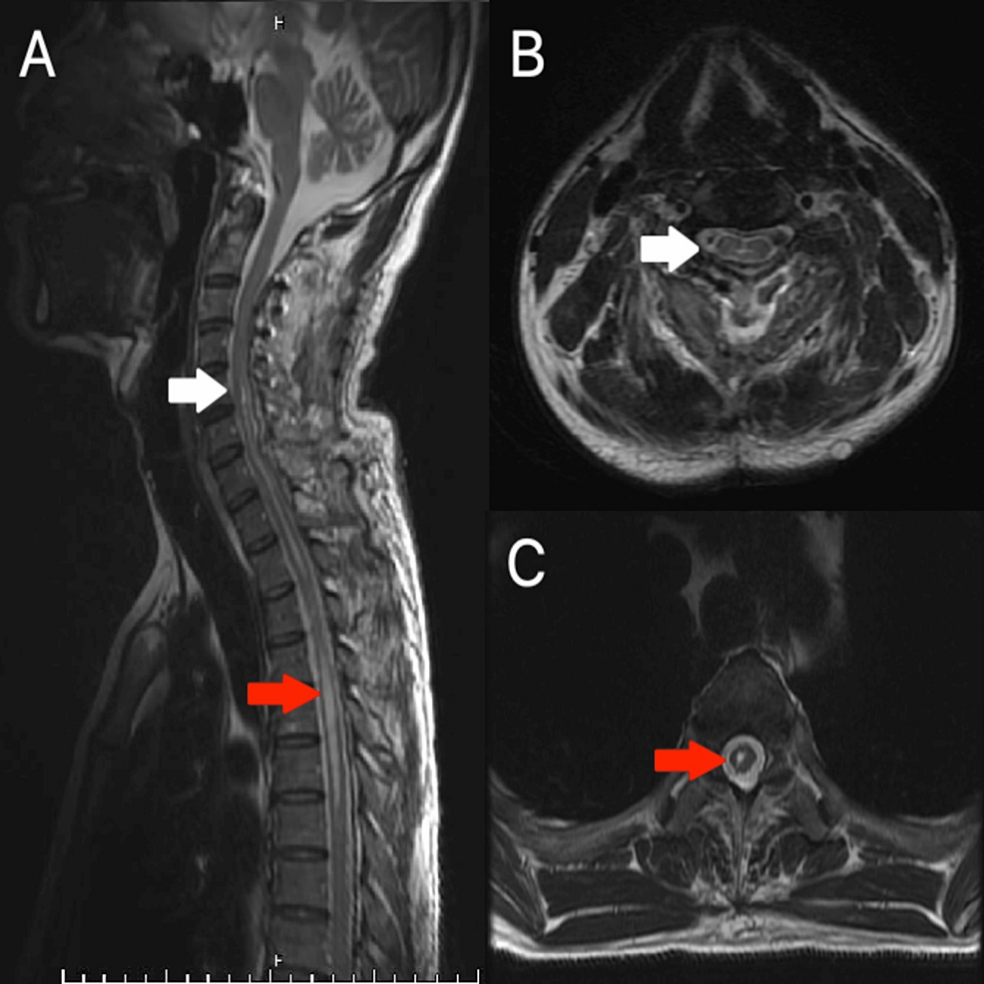
Postoperative T2-weighted MRI. The tumor is completely removed on the sagittal plane (A) and the axial plane at C5 (B, white arrow) and at the T4-T5 level corresponds to the syringomyelia (C, red arrow).

Postoperatively, the patient did not have any issues. T2-weighted sagittal MRI confirmed that the whole tumor had been removed and there was no recurrences (Figures 3A-3C).

## Discussion

Table 1 presents a review of the literature with cases of giant cervical ependymomas associated with syringomyelia.

**Table 1:** A summary of cases of giant cervical ependymomas with syringomyelia reported in the literature. CSF = cerebrospinal fluid

Author	Age	Sex	Clinical presentation	Location	syringomyelia	Surgery	Complication	McCormick score
Fourneyet al. (2004) [6]	22	M	Quadriparesis, hyperreflexia in lower limb, bilateral Babinski	C1-C7	Upper-lower	Laminectomy C1-C7	CSF fistula, meningitis	I
Barbagallo et al. (2009) [7]	25	F	Quadriparesis, ataxic gait, C4 sensory level, bilateral Babinski	C2-C5	Lower T2	Laminotomy C2-C5	No	I
Arrifin et al. (2012) [8]	30	F	Muscle atrophy upper limb areflexia, paraparesis 2/5, fecal and bladder incontinence	C2-T3	Upper-lower	Laminoplasty C3-T3	CSF fistula, surgical site infection	II
Vásquez et al. (2022) [9]	21	M	Quadriparesis patellar and bicipital hyperreflexia, bilateral Babinski and C7 sensory level, ataxic gait	C1-C7	Upper-lower	Laminotomy C1-C7	No	I

Clinical presentation

Spinal ependymomas can vary greatly. Inflammatory illness or spinal metastases have been observed in up to 60-70% of patients, in addition to benign mechanical discomfort. According to some studies, 10% of patients experience radicular pain. Due to the central structure of these tumors, touch and pain perceptions are typically the first to be impacted. Sphincter dysfunction is uncommon, with the exception of cone or cauda equina tumors. Although uncommon, tumor bleeding can sometimes result in acute neurological deterioration. Due to the possibility of neurological damage, spinal cord injuries are difficult to treat, which is further worsened if they involve giant tumors. Better functional outcomes are now feasible because of the advancement of microsurgical methods and medical technology [10].

MRI using contrast material is used to confirm the diagnosis. In almost 75% of instances, the cervical spine is the area that is most usually impacted. Our patient experienced the related lesion known as syringomyelia. Syringomyelia resolved post-surgery in all previously documented instances, including our own. A slow-growing spinal cord tumor, changes in vascular density, changes in intramedullary pressure, and degenerative changes with vessel wall hyalinosis are some of the pathomechanisms of syringomyelia [11]. It was also shown in our case that subsequent compression of lengthy spinal tracts, neurons, and artery walls causes degenerative and ischemia necrotic modifications in the tumor and glial stem below the tumor.

Degenerative cysts and tumor ischemic-related syringomyelia after ependymoma may occur as a result of disruption of the blood-brain barrier, which is supported by microcirculation impairment [11].

WHO classification and pathological examination

The term ependymoma, as used by the WHO, refers to a class of tumors that are histologically heterogeneous and include cellular, papillary, clear-cell, and tanycytic subtypes [7]. Two examples of GCE of the filum terminale were initially reported by Zec et al. [4] in 1996.

The histological grading of ependymomas has been controversial, given the large spectrum of anaplastic ependymoma prevalence (7-89%), showing how difficult it is to come to an agreement on a histological classification scheme [12]. The histopathological criteria for classifying an ependymoma as benign or malignant is one of the causes of inconsistent outcomes. A diagnosis of GCE of the cervical spinal cord was obtained after taking into account the immunohistochemical and morphological characteristics. The most accessible and valuable tool for differential diagnosis is immunohistochemistry. GCE is negative for synaptophysin and Melan A while positive for GFAP, vimentin, S-100, and CD99. The most crucial antibody for ruling out further soft-tissue malignancies is GFAP.

However, a significant shift from the traditional histomorphological categorization to a classification of 10 various types based on anatomical location and genetic characteristics has recently occurred with the 2021 revision of the WHO classification of CNS malignancies. The general idea of whether a tumor belongs to the ependymoma family or not has altered because of the development of these novel molecular kinds [13].

The four distinct tumor types that make up ependymal tumors of the spinal cord are spinal ependymoma (SP-EPN), spinal ependymoma with MYCN amplification (SP-MYCN), myxopapillary ependymoma (MPE), and subependymoma (SE). The spinal location of SP-EPN and the lack of MPE or SE morphological characteristics serve as diagnostic criteria. The most frequent location of SP-EPN after localization in the thoracic and lumbar spine is the cervical spine [14]. The *neurofibromatosis type 2* (*NF2*) gene is situated on chromosome 22q, where the majority of SP-EPN have chromosomal deletions. The tumor predisposition syndrome is brought on by *NF2 *gene mutations. Recently, SP-MYCN, a new and clinically aggressive type of ependymoma was discovered. It frequently displays early metastases, rapid recovery after relapse, leptomeningeal dissemination, and inadequate response to multimodal therapeutic strategies [15]. Spinal localization and the presence of MYCN amplification serve to identify SP-MYCN. Only a few instances have been recorded thus far [15-18]. SP-MYCN has a high recurrence rate of 75-100% [16,18], and a case study of 13 tumors indicated a median progression-free survival of 17 months [15]. The discovery of this particularly aggressive tumor form among spinal ependymomas, which are often more benign, highlights the significance of carefully reviewing current tumor classifications by genetic and clinical tests.

Treatment

The optimal treatment strategy for cervical ependymomas is total excision accompanied by intraoperative neurophysiological monitoring, frequently somatosensory evoked potentials. There are several ways to treat ependymomas. A cervical laminectomy was utilized to treat the tumor in four of the cases that were reported, while hemilaminectomy, hemilaminoplasty, and cervical laminoplasty were all performed in one case. A broad C2-T1 cervical laminectomy was performed in this instance. As in our instance, neurophysiological monitoring was employed throughout the operation without any modifications.

A precise plane between the tumor and the spinal cord, which is the ultimate goal, could not be found in our case; therefore, only 90% of the tumor was removed. Ependymomas have a plane that is well-differentiated from healthy nerve tissue, which makes complete resection possible. Cerebrospinal fluid fistulas are the most often documented complication, with two occurrences recorded, one of which was linked to meningitis and the other to a surgical site infection [19].

GCE has a good prognosis [4] because there is no connection between it and aggressive conduct. These histological characteristics are consistent with the clinical result in the current case. Increasing neurological recovery has been seen during follow-up, and an MRI scan has not revealed any indication of tumor recurrence despite the absence of adjuvant therapy, such as radiation. According to Ho et al. [20], the absence of any disease has been linked to the low Ki-67 labeling index. Ki-67 is another prognostic marker for ependymomas.

## Conclusions

Giant cervical ependymomas with syringomyelia might be completely removed without any negative effects on the nervous system or tumor recurrence. Additionally, in our patient, histology and immunohistochemical findings supported the diagnosis of a low-grade GCE together with a low Ki-67 labeling index, providing a favorable prognosis. The 2021 revision of the WHO classification states that discovering MYCN amplification for malignant alterations must be taken into account concurrently.
